# Biological control of *Aedes* mosquito larvae with carnivorous aquatic plant, *Utricularia macrorhiza*

**DOI:** 10.1186/s13071-020-04084-4

**Published:** 2020-04-21

**Authors:** Jannelle Couret, Marco Notarangelo, Sarashwathy Veera, Noah LeClaire-Conway, Howard S. Ginsberg, Roger L. LeBrun

**Affiliations:** 1 Department of Biological Sciences, University of Rhode Island, Woodward Hall, 9 East Alumni Ave, Kingston, USA; 2 U.S. Geological Survey Patuxent Wildlife Coastal Field Station, Kingston, USA; 3 Department of Plant Sciences and Entomology, University of Rhode Island, Kingston, USA

**Keywords:** Bladderworts, *Utricularia*, Biological control, Mosquitoes, *Aedes*

## Abstract

**Background:**

Biological controls with predators of larval mosquito vectors have historically focused almost exclusively on insectivorous animals, with few studies examining predatory plants as potential larvacidal agents. In this study, we experimentally evaluate a generalist plant predator of North America, *Utricularia macrorhiza*, the common bladderwort, and evaluate its larvacidal efficiency for the mosquito vectors *Aedes aegypti* and *Aedes albopictus* in no-choice, laboratory experiments. We sought to determine first, whether *U. macrorhiza* is a competent predator of container-breeding mosquitoes, and secondly, its predation efficiency for early and late instar larvae of each mosquito species.

**Methods:**

Newly hatched, first-instar *Ae. albopictus* and *Ae. aegypti* larvae were separately exposed in cohorts of 10 to field-collected *U. macrorhiza* cuttings. Data on development time and larval survival were collected on a daily basis to ascertain the effectiveness of *U. macrorhiza* as a larval predator. Survival models were used to assess differences in larval survival between cohorts that were exposed to *U. macrorhiza* and those that were not. A permutation analysis was used to investigate whether storing *U. macrorhiza* in laboratory conditions for extended periods of time (1 month *vs* 6 months) affected its predation efficiency.

**Results:**

Our results indicated a 100% and 95% reduction of survival of *Ae. aegypti* and *Ae. albopictus* larvae, respectively, in the presence of *U. macrorhiza* relative to controls within five days, with peak larvacidal efficiency in plant cuttings from ponds collected in August. *Utricularia macrorhiza* cuttings, which were prey-deprived, and maintained in laboratory conditions for 6 months were more effective larval predators than cuttings, which were maintained prey-free for 1 month.

**Conclusions:**

Due to the combination of high predation efficiency and the unique biological feature of facultative predation, we suggest that *U. macrorhiza* warrants further development as a method for larval mosquito control.
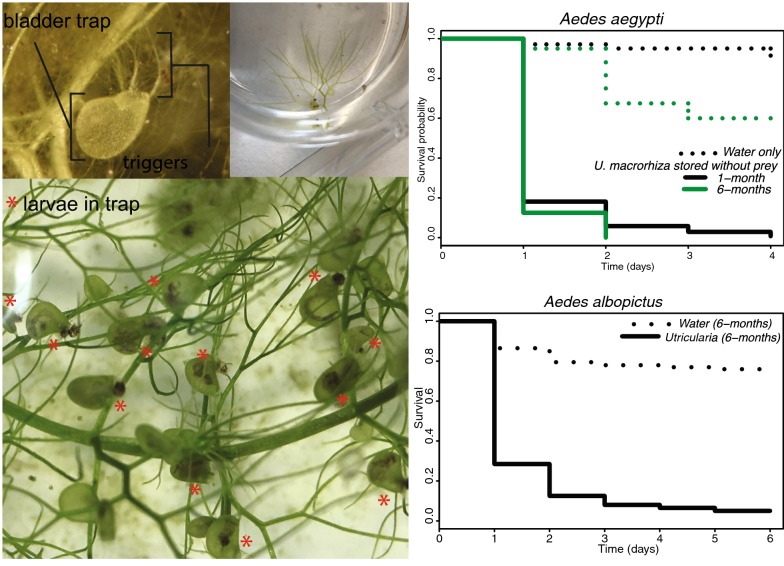

## Background

The control of larval mosquitoes with predators and other biological agents has been widely recognized as a promising strategy that can reduce negative environmental impacts associated with chemical control [1, 2]. Several diverse animal taxa have been explored as biological controls of larval mosquitoes including larvivorous fish, amphibians, and aquatic insects such as odonates and even larvae of certain mosquito species [3]. The advantages and disadvantages to each predator species are a function of prey specificity, larvacidal efficiency, and ease of management of applications for sustained periods and across the various habitats of mosquito vector species [4, 5].

Larvivorous fish have successfully controlled larvae in the genus *Anopheles* in a variety of habitats worldwide [6–11], though they have been less successful in the control of *Aedes* species [12]. This success is largely attributed to the high predation rates of species such as the mosquito fish, *Gambusia affinis* and *G. holbrooki* [13]. The disadvantage of mosquito fish is that with repeated introductions to aquatic habitats for mosquito control, there has been little consideration of their impacts on the ecosystem [14], and they have become invasive in pristine aquatic habitats [15]. Invasive mosquito fish impact native fish through indirect competition for resources [15–18] and direct competition by biting [19]. Other species of catfish have been assessed in domestic water containers with high demonstrated larvacidal efficacy for *Aedes* mosquitoes [20]. Domestic containers are not sustainable habitat for these fish and they must be replenished, a limitation of the overall feasibility of larvivorous fish for sustained control [5].

There are several options for arthropod predator controls of mosquito larvae that have been explored. Mosquitoes of the genus *Toxorhynchites* have been identified as predators of other larvae [21]. Their distribution largely overlaps with that of Aedini disease vectors [22, 23] and they colonize otherwise cryptic breeding sites that are difficult to reach for control. Field applications demonstrate limited success [24, 25] or even have resulted in an increase in prey density [26–28]. Releases of nymphal dragonflies and damselflies of Odonata as alternative predators have had mixed success [29, 30]. Unlike *Toxorhynchites* [19], odonates are generalists and can cover a wide range of habitats [31–33]. Past studies have reported promising predation rates [34–36] even in container habitats [37]. Similarly, copepods of the genus *Mesocylops* have shown promising results with regards to control of the yellow fever mosquito (*Aedes aegypti*). In Vietnam, community-involved releases have resulted in local eradications of *Ae. aegypti* from 40 non-urban communities [38, 39]. Overall however, successful applications of odonates and copepods are limited in number in part because of the difficulty in maintaining large stocks capable of supporting repeated releases in order to sustain biological control [3, 38, 40–43].

Amphibian tadpoles have demonstrated high larvacidal efficiency, although their predatory efficiency of mosquito larvae has not been estimated in the presence of alternative prey sources [5]. Several disadvantages to tadpoles for biocontrol of *Aedes* species have been noted, including low survival in small containers, the influence they exert on ecosystems, and the caution needed when considering introductions either in the low likelihood of success or in introducing an invasive species.

**Fig. 1:**
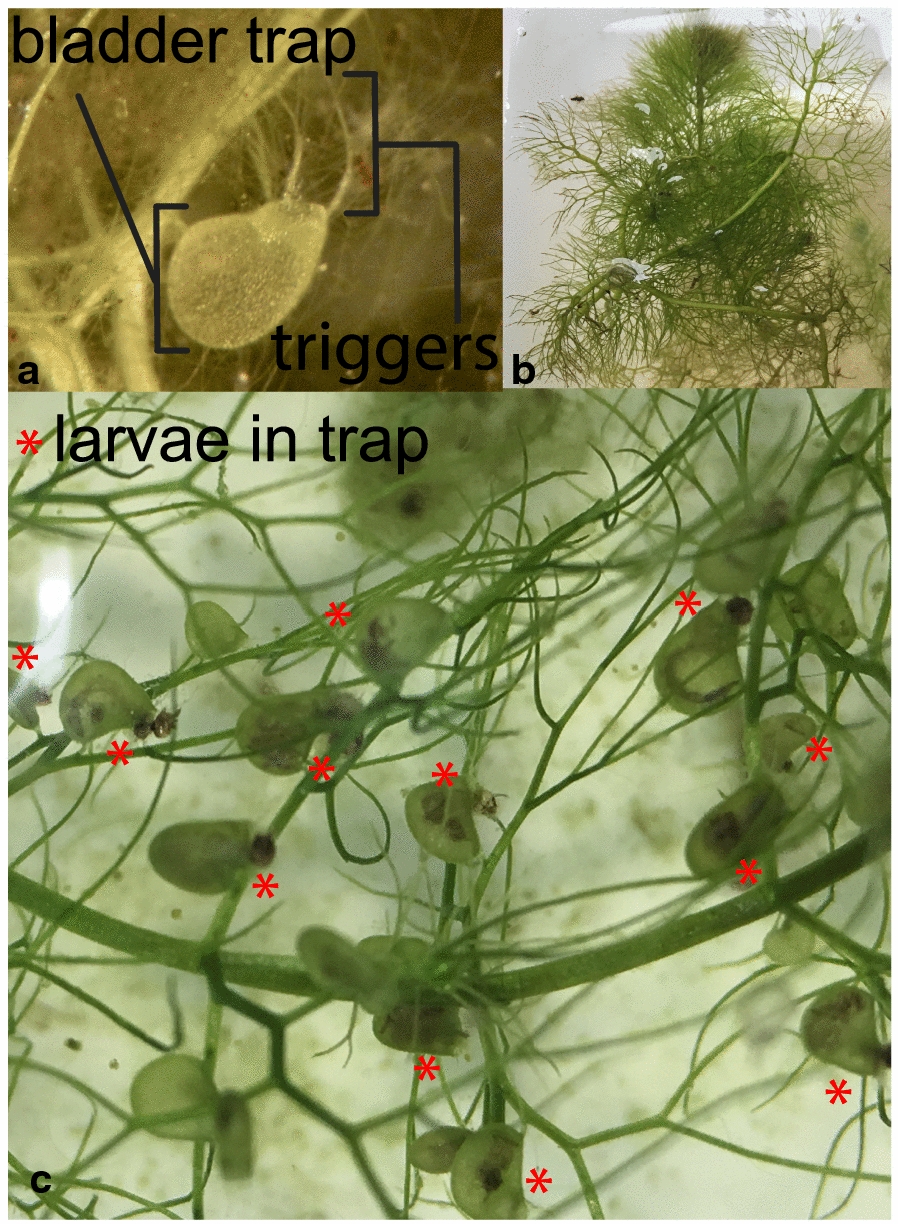
*Utricularia macrorhiza* is pictured maintained indoors in small containers in a close up view of a single bladder with trap chamber and trigger appendages labeled (**a**), expanded view of the plant cutting (**b**) and with *Utricularia macrorhiza* close up with bladders on stems digesting *Aedes aegypti* larvae indicated with red asterisks (**c**)

An understudied predator-prey association that merits exploration for biological control is that between aquatic plants in the genus *Utricularia* and mosquito larvae (Fig. 1a–c). Darwin & Darwin [44] first described the ability of *Utricularia vulgaris* to capture and asphyxiate insect larvae using lentil-shaped bladders. Bladderworts have been described as effective suction feeders of a variety of zooplankton, rotifers, protozoans, *Daphnia* and even small fish fry [45]. The biological control properties of the plant were noted and described by Matheson [45] and Twinn [46]. Despite this, the application of bladderworts as a biological control of mosquito larvae has been relatively unrecognized and understudied in recent years. Recent reviews of biological control tools for mosquito larvae excluded *Utricularia* [47] even when focusing on control with larvacidal predators [5] or alternative strategies [48]. Estimates of predation capabilities of bladderworts for mosquito larva are limited, with a notable exception. *Utricularia macrorhiza* (commonly referred to as *U. vulgaris* in North American’s literature prior to Taylor [49]) was observed to have high rates of predation on *Culex pipiens* larvae, ranging between 50–100% [50]. It has since been suggested that using bladderwort as a biological control strategy may be of limited value because of the abundance of alternative prey sources in the natural habitats of *Culex pipiens* [50–52]. These studies have centered on mosquito species that develop in permanent and temporary pools with large volumes of water. There is evidence to suggest that several *Utricularia* predators may thrive outside of their natural habitat [46, 50, 53, 54], and thus may be applied to the control of container-breeding species.

*Utricularia macrorhiza* is widely distributed in North America [55] but has yet to be explored in small water containers such as those utilized by *Aedes* mosquitoes for larval development. In this study, we explore the potential for aquatic bladderworts in the genus *Utricularia* (Lentibulariaceae) as predators of *Ae. aegypti* and *Ae. albopictus*. These species have a habitat preference for small man-made containers that are naturally prey-limited [12, 56–58], and this preference has been a driving feature of their expansion through urban areas [59, 60].

We first sought to determine if plant cuttings could survive when displaced from their natural habitats for lengthy periods of time and placed in small man-made containers typically inhabited by *Ae. aegypti* and *Ae. albopictus* mosquitoes. We hypothesized that *U. macrorhiza* would readily predate mosquito larvae regardless of species and larval stage, and effectively reduce mosquito laboratory populations through direct impacts on survival during the larval stages. We tested this hypothesis in no-choice, laboratory rearing experiments, and estimated daily predation efficiency of plant cuttings of standardized bladder density.

Both *Ae. aegypti* and *Ae. albopictus* are historically and currently important vectors of pathogens including dengue, yellow fever, Zika and chikungunya viruses [61, 62], and although few autochthonous cases have been noted recently in their USA range, the distribution and abundance of these vectors is resurging in recent years [63]. The rise of insecticide resistance in natural mosquito populations [64, 65], combined with the discovery of non-target effects of chemical pesticides on other species, including humans [66–68] underscores the need to develop alternative, eco-friendly strategies for the management strategies for these vectors.

## Methods

### Mosquito colony conditions

Laboratory colonies of *Ae*. *aegypti* strain originating from Puerto Rico and *Ae. albopictus* colony, originating from New Orleans, LA, were maintained at 27 °C, 75% RH, with a 16:8 L:D photoperiod. Experimental larvae were hatched from the laboratory colony from generations F10-F18 and placed in experimental conditions within 24 h of hatching.

### Bladderwort collection/cultivation

**Fig. 2:**
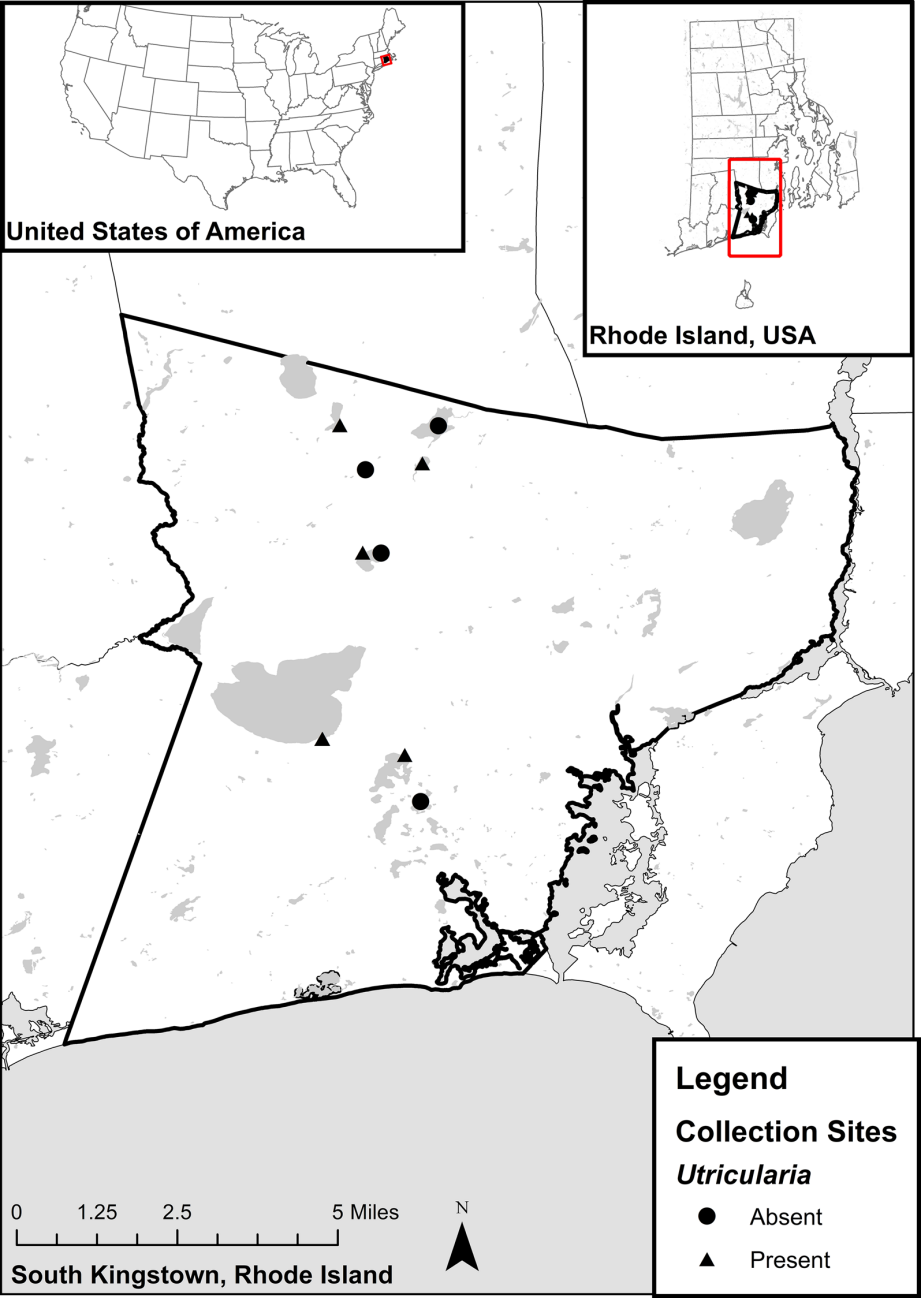
Presence and absence of *Utricularia* spp. at collection sites in Washington County, Rhode Island

Common bladderwort (*U. macrorhiza*) was collected throughout the spring, summer and fall seasons of 2017 from 6 freshwater ponds in South Kingstown, RI, USA (Fig. 2). The presence of other species in the genus *Utricularia* was noted for each pond. Whole plants and segments of approximately 30–45 cm in length were sampled from the edges of ponds by hand and transported in water to the laboratory. Strands of *U. macrorhiza* were checked and cleared of symbiotic odonates. Plants were placed in container-tubs and left to acclimate to laboratory conditions (at room temperature) for a minimum of a month before being used for experimentation. Bladderworts continuously grow bladders, which become active and decay. A constant number of bladders was therefore not feasible, but strands were chosen for the experimental period which has approximately 100 bladders in order to start the experiments with an initial bladder to larva ratio of 10:1.

### Predation of container-dwelling mosquitoes by *U. macrorhiza*

Experimental eggs were hatched in Picotap-filtered water by multiple-immersion clue. Eggs were briefly submerged and dried for three times prior to hatching to simulate oxygen fluctuation that would be typical under field conditions. We examined the survival rates of container-dwelling mosquitoes in the presence of predating *U. macrorhiza* under the conditions of 10 larvae per 500 ml of Picotap-filtered water with a 15-cm-long segment of *U. macrorhiza* with approximately 100 bladders. We recorded the survival status and developmental stage of each individual on a daily basis until death or emergence occurred. Larvae were fed every-other-day with finely ground and sieved fish-food (TetraMin Tropical Flakes, Tetra, Melle, Germany). Food was added on a per-capita basis to each cup [69] such that larvae were provided 0.06 mg/larva on day 1, 1.0 mg/larva on day 3, 1.5 mg/larva on day 5, and 1.8 mg/larva on day 7. Upon emergence, adults were transferred into 2.0 ml microcentrifuge tubes and stored at − 30 °C.

Fourteen replicates were conducted with *Ae. aegypti* larvae and plant cuttings that had been without prey for one month. Four additional replicates were conducted with *Ae. aegypti* with cuttings that had been stored in open containers in a windowsill indoors at ambient room conditions without availability of prey for 6 months. Twenty replicates were conducted for *Ae. albopictus* using cuttings of *U. macrorhiza* that had been stored 6 months without prey. Because the period without prey is known to alter the number of bladder traps in several species of *Utricularia* [70–73], we separated replicates based on the number of months the plants had been stored. However, the initial number of bladders used in experimental cups was standardized to 100 bladders. Therefore, differences observed between 1-month replicates and 6-month replicates are attributed to differences in bladder trapping activity rather than the number of bladders. For each replicate, the number of bladders per *U. macrorhiza* segment was measured less than 24 h before set-up. The cause of larval mortality was attributed to direct predation when larvae were found wholly or partially inside of bladders. When larvae were found dead outside of bladders cause of death was not noted. The experiment concluded when all *U. macrorhiza* exposed larvae either died or emerged.

We investigated whether *U. macrorhiza*, under similar laboratory conditions as previous experiments, was able to predate third- and fourth-instar *Ae. aegypti* larvae. We placed 8 replicates of ten larvae that were initially third-instar to *U. macrorhiza* segments. Over the course of the experiment several larvae molted to fourth-instar. After 24 h we recorded total survival and life stage. The aim of this experiment was to assess whether bladders were capable of trapping larger prey. Bladder size is highly variable even within *U. macrorhiza*. We estimated bladder traps used in this experiment to range from 2–4 mm in width.

### Statistical analysis

Statistical analyses were conducted in RStudio v.1.0.143 [74] using the *survival* package v.2.42-6 [75]. We estimated the effects that exposure to *U. macrorhiza* had on mosquito survival using the Cox-proportional Hazard model with an Efron approximation and Weibull function [76]. The assumption of proportional hazard was tested using Schoenfeld’s residual test [77]. Bladders predate to satiation and thus their ability to impact mosquito survival is implicitly linked with time. Thus, it was expected that these data would violate the assumption of proportional hazard. While the Mantel-Cox logrank test [78] is the most commonly used statistical method of comparison for survival curves, its usage becomes unsuitable when the hazard ratio does not remain proportional with time, as these data suggested. To account for this violation of the assumption of proportionality with the Mantel-Cox test, we instead used the non-parametric Peto & Peto modification of the Gehan-Wilcoxon test. This method remains robust even when the assumption of proportional hazard is violated [79].

## Results

**Fig. 3:**
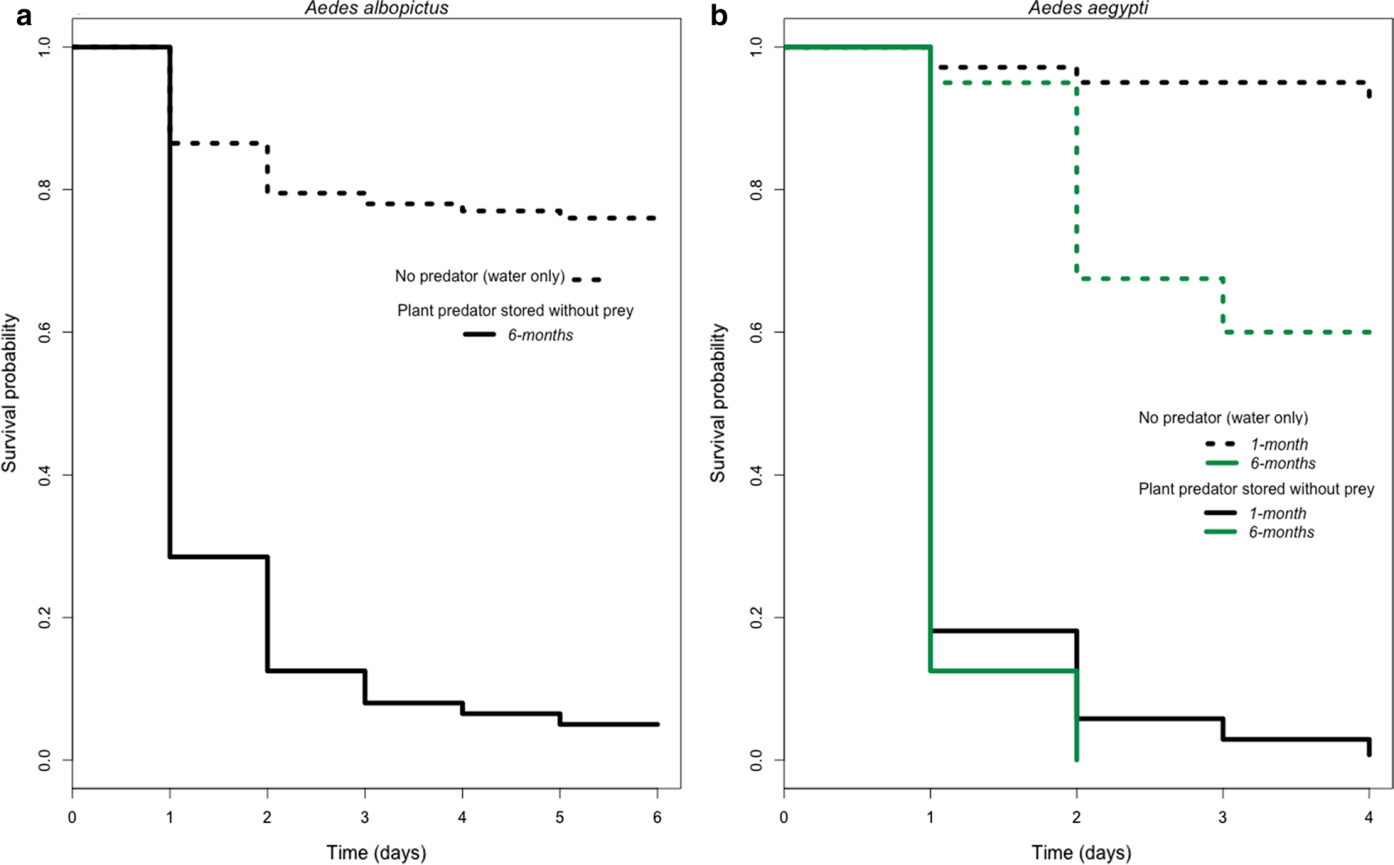
Survival probability over time (in days) of *Ae. albopictus* (**a**) and *Ae. aegypti* (**b**) in the presence and absence of *U. macrorhiza* stored without prey for six months. Dotted lines represent water-only controls. Solid lines represent experimental cups with *U. macrorhiza*. Black indicates plants were stored for 1 month without prey and green indicates plants were stored for 6 months without prey. For both figures, data are censored as of the day when the last death from predation by *U. macrorhiza* was observed

*Aedes albopictus* replicates exposed to plant predation showed a greater Cox proportional hazard than controls (Fig. 3a; Likelihood ratio test: 239.9, *df* = 1, *P* < 0.0001). There was sufficient mortality in control cups, which developed in the absence of predators, to develop Cox proportional hazard estimates (HR = 9.812, CI: 7.06–13.66, *P* < 2 × 10^−16^). In cups with the plant predator an average of 71.5% of larvae died within the first 24 h. Over the course of the next four days larvae continued to be preyed upon, with 16% of larvae dying on the second day, 4.5% dying on the third day, 1.5% dying on the fourth day, and a further 1.5% dying on the fifth day. By the end of the fifth day, 95% of all larvae coexisting with *U. macrorhiza* had died. No further deaths due to predation occurred past the fifth day. Out of the surviving individuals (*n* = 10), all but one originated from the same experimental container. A non-parametric test of survival hazards comparing predation in experimental cups *versus* treatments cups shows that predation by *U. macrorhiza* significantly reduced larval survival (*χ*^2^ = 209, *df* = 1, *P* < 1 × 10^−16^).

Similarly, the presence of the plant predator was found to significantly reduce *Ae. aegypti* survival under laboratory conditions (*χ*^2^ = 308, *df* = 3, *P* < 1 × 10^−16^, Fig. 1c). Across all replicates, the average predation efficiency was highest during the first 24 h, during which, 83.1% of larvae were found consumed within bladder traps. Within 48 h 95.5% of larvae were preyed upon. On days three and four 97.7% and 99.4% of larvae were preyed upon, respectively (Fig. 3b). By day 5 all larvae within cups with a plant predator were consumed. Having been placed within 24 h of hatching, the latest developmental stage achieved by *Ae. aegypti* larvae in the presence of predating *U. macrorhiza* was the second instar.

In addition to comparing treatments with and without the plant predator, we considered the number of months that plant cuttings sat without prey. Table 1 presents the results of a permutation model of *Ae. aegypti* larval survival that accounts for both the time plant cuttings were stored without prey (one month or six months) and treatment (presence or absence of predator) which significantly improved prediction of larval survival probability over a model of treatment alone (*F*_(1, 356)_ = 25.03, *P* < 8.87 × 10^−7^).

**Table 1:** Permutation analysis of *Ae. aegypti* survival times with treatment (presence or absence of plant predator, *U. macrorhiza*) and trial (1 or 6 months of storage time without prey for plant predator prior to experiment)

Source	*df*	Sum Sq	Mean Sq	Iterations	Significance
Trial	1	1.620	1.620	5000	***
Treatment	1	63.184	63.184	5000	***
Residuals	356	23.04	0.065		

***P < 0.001

A further experiment was conducted to determine whether *U. macrorhiza* was capable of preying upon third-instar (Additional file 1: Video 1) and fourth-instar (Additional file 2: Video 2) *Ae. aegypti* larvae. Eight replicates of 10 larvae each were placed into containers with *U. macrorhiza*. After 24 h the predation efficiency was variable from 60 to 100% consumed, demonstrating that plant predatory bladders were capable of consuming later instars (mean ± SE, 77.5 ± 4.91%).

**Fig. 4:**
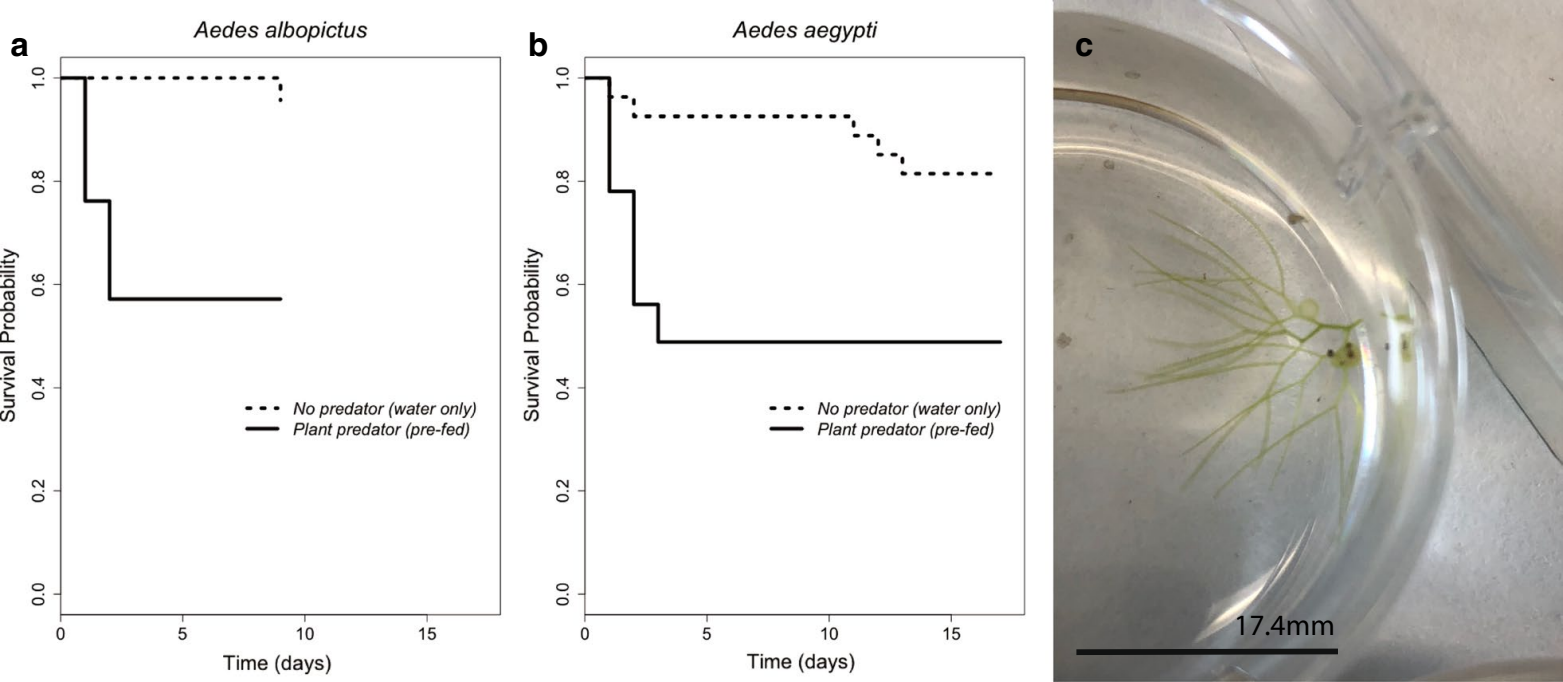
Survival probability over time (in days) of *Ae. albopictus* (**a**) or *Ae. aegypti* (**b**) with small cuttings of *U. macrorhiza* with two bladders placed in 10 ml of dH_2_0. Pre-fed signifies that bladders were provided one larva of the respective species just prior to the start of the experiment. Image of the experimental set up with the *U. macrorhiza* cutting having consumed 3 larvae consecutively (heads visible as black dots inside bladder) (**c**)

We carried out predation experiments with small cuttings of *U. macrorhiza* measuring approximately 1.25 cm, with one bladder and placed into the well of a 6-well cell-culture plate with 10 ml of water. We pre-fed the bladder with one larva and counted the number of replicates which predated a second larva of *Ae. aegypti* or *Ae. albopictus* over the course of the experiment. We found that larval environments with small cuttings of *U. macrorhiza* with even a single bladder can effectively reduce larval survival relative to conditions without the plant present (Fig. 4a, b). We also found that a one bladder under these conditions can potentially hold up 3 larvae (Fig. 4c).

## Discussion

In this study we evaluated the predation efficiency of *U. macrorhiza* in two medically important species of *Aedes* mosquitoes, finding drastic and effective reduction of daily survival for *Ae. aegypti* and *Ae. albopictus* larvae in no-choice predation experiments. The effective control of larval population for both *Ae. aegypti* and *Ae. albopictus*, suggests that *U. macrorhiza* is a viable option to explore for biocontrol of container-breeding mosquitoes even in small water volumes. Although *U. macrorhiza* survival and growth were not formally measured under prolonged laboratory conditions, through this study, we determined that the plant is a capable predator of mosquito larvae even after six months after displacement from its original habitat.

We found *U. macrorhiza* to be capable of preying on first- through third-instar *Ae. aegypti* larvae. These results are in line with previously published work [80], which suggested that *U. macrorhiza* can predate mosquitoes at three stages of development. These results were consistent when repeated at smaller water volumes. In comparison to the predation experiments at larger volumes, the survival of larvae exposed to a single bladder on small cuttings of *U. macrorhiza* is at first glance reduced. However, the bladder to larva ratio in the latter experiment was 1:1, whereas the previous experiments had a ratio of 10:1. While control of larvae at such small water volumes is impractical, these results demonstrate that small water volume is not on its own a limiting factor in the application of *U. macrorhiza*.

It is possible that *U. macrorhiza* is capable of effectively preying upon *Aedes* pupae or large fourth instars; however, the trap sizes observed under laboratory conditions were smaller than those initially collected in the field. As the metamorphic stage, pupae do not forage for food and thus might not interact as frequently with bladders as foraging larvae. However, we expect that fourth instars would be susceptible to *U. macrorhiza* predation. We observed in third instars that although bladders did not wholly consume them, they were trapped by the siphon, resulting in asphyxiation. Previous work on bladderworts shows that trap size and the ability of the bladders to capture prey is largely dependent on nutrient availability [46, 81–84]. Predation efficiency on larger prey, including fourth instars likely depends upon the environmental conditions in which it is being measured [80, 85].

Angerilli & Beirne [86] explored another *Utricularia* species, *Utricularia minor*, finding similar results that the plant is capable of eliminating *Ae. aegypti* larvae within 6 days of exposure under artificial container conditions. We found that larval *Ae. aegypti* were eliminated within four days of exposure to *U. macrorhiza*, which suggests while there may be some variation between plant species in predation efficiency, there is potential for applying several species within *Utricularia* to biological control of *Ae. aegypti*. Similarly, *Ae. albopictus* larvae were eliminated by day 5. There was one replicate exception for *Ae. albopictus*, a cup in which *U. macrorhiza* preyed on only 10% of developing larvae. We attribute the low survival in this replicate to the readily observable poor quality of the cutting used, with greater numbers of senescent bladders. Senescent bladders are known to continue to photosynthesize but do not fire as often or effectively capture prey [87]. Bladders regularly are produced and senesce on cuttings; it is unclear why, but we observed this replicate lost many bladders in the course of the experimental period. The experimental results showed some differences in predation between the two species considered (Fig. 3). Notably, plant predation was sufficient to eliminate larvae prior to the number of days typically needed for larvae to complete larval development.

Bladderworts can exist for extended periods without prey, adaptively shift to carnivory, and increase predatory efficiency as prey density increases. When plants are maintained in the absence of prey for long periods, it can impact the number of bladders [70–73]. Englund & Harms [88] demonstrated that the investment in predatory biomass (bladders) increases at high prey densities. Subsequently as prey populations dwindle with predation, nutrient enrichment in the plant results in a shift away from carnivory and toward photosynthesis. Indeed, bladderworts exhibit the highest rates of photosynthesis among submerged plants [89]. This suggests that long-term maintenance of nutrient poor conditions is essential to stimulate bladder production [90]. Our results indicated that extended periods without prey did not negatively impact the ability of all but one experimental cup to predate larvae of *Ae. aegypti* and *Ae. albopictus*. Facultative predation, and plasticity in energy allocation toward different growth strategies differentiates bladderworts from other animal predators currently in use for biological control. While not all oviposition sites of *Ae. aegypti* or *Ae. albopictus* will be practical or appropriate for control by a photosynthetic plant, we expect *U. macrorhiza* to be appropriate for a variety of sunlit water storage vessels which individuals are unable or unwilling to empty.

It is possible that bladderworts may be used alongside other chemical and biological control tools. Bladderworts have not yet been explored in conjunction with other control agents, but have been found to be highly resistant to certain insecticides, pesticides and herbicides [91–93]. Bladderworts are not expected to be vulnerable to the most commonly deployed larvacidal biological control measures, *Bacillus thuringiensis* var. *israelensis* (Bti) or *Bacillus sphaericus*, due the bacteria’s specificity to larvae of some Diptera [94, 95]. Indeed, water pools containing *Utricularia* plants are preferred as oviposition sites by damselflies and other mosquito predators [96, 97], suggesting that introducing *Utricularia* into novel containers may indirectly affect mosquito populations by aiding the natural predators of container-breeders to establish in these otherwise cryptic environments [98–104]. These results suggest the potential for bladderworts to be useful and merit further experiments to explore the impacts of combination with other biological control methods.

The effectiveness of a predatory biological control agent depends on a variety of factors that include the biological features of predators and predation efficiency as well as aspects of the management of stocks for biocontrol applications. Biological features relevant to control of larvae include habitat overlap, prey specificity, predatory efficiency, and population dynamics and auto-reproduction. Feasible management of predator populations for biological control include ease of growing and maintaining stock, overlap in distribution between predator and prey and survival in prey habitats, auto-reproduction for sustained control, and the cost-effectiveness of the biocontrol measure [105]. One advantage of aquatic bladderworts as a biocontrol is their extended period of efficacy. Previous field experiments have found various *Utricularia* plants to be effective at controlling macro-invertebrate preys throughout the summer season [106]. The plants are most predacious in July and August [106], suggesting that their main period of efficacy coincides with that of multivoltine mosquito vectors [107, 108]. The synchrony in seasonality between aquatic bladderworts and mosquito vectors suggests that early releases of the plants may be sufficient to inhibit the development of vectors within accessible container habitats during peak season. In contrast, applications of other common biocontrol measures such as *Bacillus thuringiensis* var. *israelensis*, *Toxorhynchites*, or odonates generally require two or more seasonal applications to be effective [43, 109–111].

As bladderworts are globally widely distributed generalist predators across every continent except Antarctica [55]. All *Ae. aegypti-* and *Ae. albopictus*-colonized continents have *Utricularia* plant species that are suitable for vector-control. The plant here studied, *U*. *macrorhiza*, is broadly distributed in North America, Central America and North Asia [55], while Europe and Northern Africa are colonized by a related species also known to predate mosquitoes, *U. vulgaris* [55, 80]. In Central Africa, *Utricularia radiata* has recently been identified as a potential biocontrol [54]. To the best of our knowledge, no bladderworts have been examined for their biocontrol properties in South America and Australia, but both continents are considered “hot spots” with regards to *Utricularia* diversity [112], with various studies documenting the plants’ diets [103, 113], suggesting that finding local alternatives to *U. macrorhiza* is plausible. The wide distribution of native *Utricularia* species signifies that this method need not rely on the introduction of non-native species to control mosquitoes in a given area.

Environmental impacts of the use of *U. macrorhiza* or other *Utricularia* species should be considered in comparison to the current methods commonly used, both biological and chemical. The proposed application to control *Aedes* vector species is limited to container-breeding sites rather than natural aquatic systems. The specificity of the bladderworts, predating only aquatic organisms within the container, reduces the impact on non-target organisms. Further, as these are freshwater predators, plant cuttings are not expected to have a negative impact on ecologically beneficial pollinators [114].

## Conclusions

This study provides insights into the potential for local predacious bladderworts to work as biological controls of container-breeding mosquitoes, especially in the peri-domestic environment. As an alternative to chemical controls that harm non-target insects, *Utricularia* produces emergent flowers that are pollinated by insects [115], and thus can supply floral resources for bees. Integrated vector management strategies can reduce impacts on non-target insects, pollinators in particular [114], and any novel method for biocontrol must be evaluated for efficacy in mosquito control as well as its impact on beneficial insects. Future studies should evaluate the feasibility, practicality, and effectiveness of biological control of *Aedes* larvae using *U. macrorhiza* and additional *Utricularia* species under a variety of field conditions. Similarly, interactions between *Utricularia* plants and other common animal predators utilized for biocontrol should be evaluated to assess interactions that could impact the incorporation of *Utricularia* into integrated vector management strategies.

## Supplementary information

**Additional file 1: Video S1.** Third-instar *Ae. aegypti* larva captured by an *Utricularia macrorhiza* bladder under artificial container conditions.

**Additional file 2: Video S2.** Fourth-instar *Ae. aegypti* larva captured by an *Utricularia macrorhiza* bladder under artificial container conditions.

## Data Availability

The datasets of the study are available from the corresponding author upon reasonable request. Plants were collected in Rhode Island with permission through a Type I Scientific Collector’s Permit issued by the Rhode Island Department of Environmental Management.
